# Mutation D614G increases SARS-CoV-2 transmission

**DOI:** 10.1038/s41392-021-00502-w

**Published:** 2021-03-01

**Authors:** Prerna Arora, Stefan Pöhlmann, Markus Hoffmann

**Affiliations:** 1 Infection Biology Unit, German Primate Center, Göttingen, Germany; 2 Faculty of Biology and Psychology, Georg-August-University Göttingen, Göttingen, Germany

**Keywords:** Microbiology, Infectious diseases

It has been unclear why SARS-CoV-2 with a D614G mutation in the spike protein became dominant early in the COVID-19 pandemic. A recent study by Hou et al. published in *Science* shows that D614G increases SARS-CoV-2 spread in cultured human nasal epithelium and enhances transmission in a hamster model.^1^

The COVID-19 pandemic has claimed more than two million lives and has wreaked havoc on the global economy. The causative agent, SARS-CoV-2, is believed to have been transmitted from an animal reservoir, most likely bats, to humans and such a zoonotic spillover is not unprecedented. In 2002, SARS-CoV crossed the species barrier and caused the SARS epidemic that was associated with over 8000 cases. SARS-CoV and SARS-CoV-2 share several biological commonalities, particularly with regard to viral entry into target cells. The spike (S) proteins of both viruses use the cellular protein ACE2 as a receptor and the cellular protease TMPRSS2 for S protein priming. However, there are also major differences. The most prominent one is the efficient replication of SARS-CoV-2 in the upper respiratory tract and its high human-to-human transmissibility that was not observed for SARS-CoV.

The S protein is a major determinant of coronavirus tropism and likely also intraspecies transmissibility. Interestingly, SARS-CoV-2 with mutation D614G in the S protein became dominant early in the pandemic^2^ and D614G is present in variant VOC 202012/01 (also known as B.1.1.7), which has become highly prevalent in Southeast England. D614G was associated with increased viral loads in the upper respiratory tract^2^ and augmented transmissibility of D614G-bearing viruses has been suspected. However, increased transmissibility remained to be demonstrated and the underlying mechanism remained to be explored. Moreover, it has been suggested that D614G might be associated with increased S protein stability and particle infectivity but might have little impact on antibody-mediated neutralization.^3,4^ However, confirmation with authentic SARS-CoV-2 was largely lacking.

**Fig. 1:**
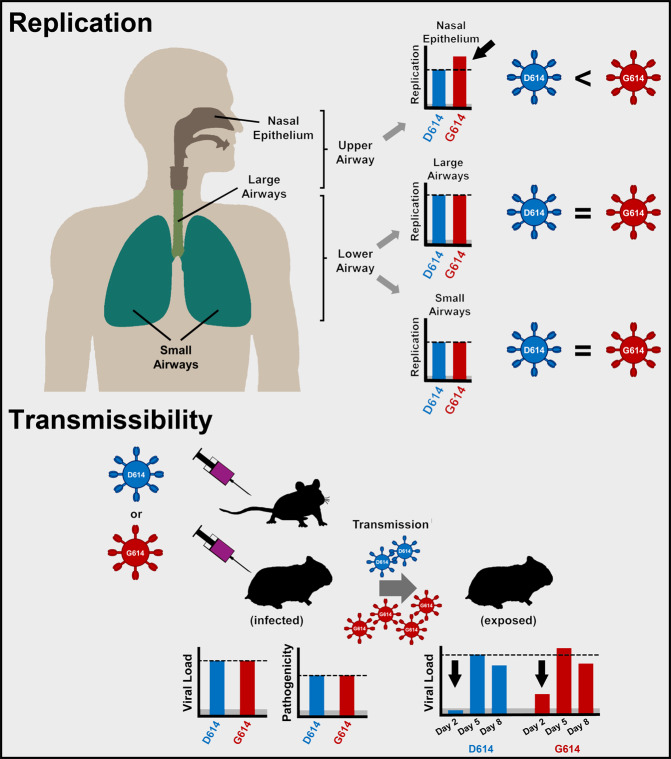
Mutation D614G in the SARS-CoV-2 spike protein augments transmission. Viruses with the amino acid substitution D614G show increased replication capacity in the human nasal epithelium but not small and large airways ex vivo. Replication of G614 virus in the nasal epithelium of experimentally inoculated hamsters was not increased but the G614 virus was transmitted faster to recipient animals as compared to the D614 virus

A seminal study by Hou et al. provided important insights into the role of D614G in SARS-CoV-2 infectivity, spread, pathogenesis, and transmission.^1^ The group employed reverse genetics to generate SARS-CoV-2 harboring mutation D614G. The G614 virus entered cell lines with higher efficiency than the D614 virus but this did not translate into increased multicycle replication for at present unclear reasons.^1^ Different findings were obtained with primary respiratory epithelium ex vivo: G614 virus replicated to higher titers than D614 virus in human nasal, but not small or large airway epithelium and outcompeted D614 virus (Fig. 1). Remarkably, no differences between D614 and G614 viruses were observed for particle shape, S protein incorporation, and proteolytic processing.^1^ In mouse and hamster models for SARS-CoV-2 infection of humans, no appreciable differences in D614 and G614 virus replication and pathogenesis were observed although hamsters infected with G614 virus lost slightly more weight than D614 virus-infected animals.^1^ In contrast, the G614 virus was transmitted faster between hamsters than the D614 virus (Fig. 1), indicating that D614G specifically confers higher transmissibility by an as yet not fully understood mechanism.^1^

Studies with pseudotyped particles had initially suggested that D614G increases cell entry.^2,4^ These findings have been confirmed with authentic SARS-CoV-2 in this study^1^ and might be explained by G614 S trimers being more frequently in a conformation suitable for ACE2 binding as compared to D614 trimers.^3^ The increased viral replication in human nasal epithelium ex vivo is in keeping with the results of a recent study^5^ and with the increased viral load in the upper respiratory tract of human patients infected with the G614 virus.^2^ The increased viral replication of the G614 virus in the upper respiratory tract may augment human-to-human transmissibility and account for the global dominance of viruses bearing D614G but the underlying mechanism remains elusive. One can speculate that G614 might augment the usage of attachment-promoting factors or TMPRSS2-related S protein-activating proteases mainly expressed in the upper respiratory tract. In addition, D614G may be associated with increased particle stability.^5^

The work by Hou et al. demonstrates for the first time that although the D614G exchange has little if any impact on pathogenesis it promotes transmission.^1^ The replication of the G614 virus in the nasal epithelium of hamsters used for transmission experiments was not increased relative to the D614 virus (Fig. 1). However, a separate study reported slightly augmented G614 virus levels in nasal epithelium of infected hamsters and higher specific infectivity of the G614 virus.^5^ Similarly, the G614 virus outcompeted the D614 virus in the nasal epithelium of infected animals.^5^ Hou et al. moved these analyses a critical step forward by demonstrating that the G614 virus is transmitted faster than the D614 virus.^1^ Thus, contact animals exposed to the G614 virus showed viral replication in the nasal epithelium earlier than animals exposed to the D614 virus (Fig. 1), formally demonstrating for the first time that D614G augments transmission.

Concerns have been raised that the global dominance of the G614 virus may compromise the efficacy of vaccines, which have been generated using the D614 virus. Hou et al. demonstrate that this concern is unfounded.^1^ Thus, no appreciable difference in the neutralization of D614 and G614 virus by sera from COVID-19 patients or monoclonal antibodies was observed.^1^ A separate study reached similar conclusions,^3^ while another one suggested that G614 viruses might be moderately more susceptible to antibody-mediated neutralization.^5^ While these minor discrepancies might be due to differences in the experimental systems used, both studies clearly indicate that G614 will not compromise SARS-CoV-2 control by the humoral immune response.

More information is needed to fully understand how D614G promotes transmission. Thus, the molecular mechanism underlying increased replication of G614 viruses in nasal epithelial cells remains largely open and we offered potential explanations above. Further, the interplay of D614G with other mutations in the S protein remains to be explored. Finally, it is important to better link cell culture and clinical data to understand how and why D614G-bearing viruses drive the pandemic.

In sum, Hou et al. as well as previous studies show that SARS-CoV-2 can acquire mutations that might increase viral spread in the human population and it will be imperative to monitor the circulating viruses for such mutations.
